# CAPRIB: a user-friendly tool to study amino acid changes and selection for the exploration of intra-genus evolution

**DOI:** 10.1186/s12864-020-07232-3

**Published:** 2020-11-26

**Authors:** Juan F. Guerra Maldonado, Antony T. Vincent, Martin Chenal, Frederic J. Veyrier

**Affiliations:** Institut national de la recherche scientifique, Centre Armand-Frappier Santé Biotechnologie, Bacterial Symbionts Evolution, Laval, Québec Canada

**Keywords:** Software, Molecular evolution, Protein evolution, Phylogeny, *Mycobacterium*

## Abstract

**Background:**

The evolution of bacteria is shaped by different mechanisms such as mutation, gene deletion, duplication, or insertion of foreign DNA among others. These genetic changes can accumulate in the descendants as a result of natural selection. Using phylogeny and genome comparisons, evolutionary paths can be somehow retraced, with recent events being much easier to detect than older ones. For this reason, multiple tools are available to study the evolutionary events within genomes of single species, such as gene composition alterations, or subtler mutations such as SNPs. However, these tools are generally designed to compare similar genomes and require advanced skills in bioinformatics. We present CAPRIB, a unique tool developed in Java that allows to determine the amino acid changes, at the genus level, that correlate with phenotypic differences between two groups of organisms.

**Results:**

CAPRIB has a user-friendly graphical interface and uses databases in SQL, making it easy to compare several genomes without the need for programming or thorough knowledge in bioinformatics. This intuitive software narrows down a list of amino acid changes that are concomitant with a given phenotypic divergence at the genus scale. Each permutation found by our software is associated with two already described statistical values that indicate its potential impact on the protein’s function, helping the user decide which promising candidates to further investigate. We show that CAPRIB is able to detect already known mutations and uncovers many more, and that this tool can be used to question molecular phylogeny. Finally, we exemplify the utility of CAPRIB by pinpointing amino acid changes that coincided with the emergence of slow-growing mycobacteria from their fast-growing counterparts. The software is freely available at https://github.com/BactSymEvol/Caprib.

**Conclusions:**

CAPRIB is a new bioinformatics software aiming to make genus-scale comparisons accessible to all. With its intuitive graphical interface, this tool identifies key amino acid changes concomitant with a phenotypic divergence. By comparing fast and slow-growing mycobacteria, we shed light on evolutionary hotspots, such as the cytokinin pathway, that are interesting candidates for further experimentations.

## Background

Bacteria are ubiquitous in nearly any given environment because of their ability to quickly evolve and adapt. As for other living beings, bacteria evolve through genetic changes that allow them to increase their fitness in response to changing environments and hosts. Thanks to recent advances in sequencing technologies (reviewed in [1, 2]), we are now able to easily sequence bacterial genomes from diverse ecological niches [3, 4], allowing us to unravel much more clearly than before the evolutionary mechanisms that shaped bacterial adaptation.

Gene deletions and insertions are two major genetic events that have been linked with bacterial evolution. Supporting this statement, it has been shown in the *Neisseriaceae* family that the deletion of a specific gene in a bacilli-shaped ancestor led to the transition towards a coccoid shape, which is thought to help immune evasion in the human nasopharynx [5]. On the other hand, horizontal gene transfer (HGT) is a major evolutionary force in prokaryotes through gene acquisition [6]. The *Mycobacterium* genus is a perfect evidence of beneficial insertions by HGT, with several genes acquired being necessary for the cell’s metabolism and virulence [7–10]. A recent study suggests that the unique cell envelope of bacteria members of the order *Corynebacteriales*, such as *Mycobacterium tuberculosis*, might be the result of a stepwise acquisition of multiple genes [11], supporting the importance of these events in bacterial evolution. Given the increase of genomic studies, a considerable number of tools have been developed in recent years to investigate the evolution of the gene repertoire, such as MycoHIT [8], SaturnV [12], GET_HOMOLOGUES [13], and Roary [14].

Although gene acquisition and deletion can reveal insights on some evolutionary processes in bacteria, they are not sufficient to completely explain bacterial adaptation. Other subtler genetic events, like single nucleotide substitutions or single amino acid (AA) changes, are necessary for evolution. These point mutations arise naturally from replication errors and are therefore believed to be much more frequent than gene rearrangements [15]. In the *Mycobacterium tuberculosis* complex (MTBC), at the species scale, thousands of single-nucleotide polymorphisms (SNPs) have been identified in numerous virulence genes [16]. In addition, multiple studies have also shown the importance of AA changes in the evolution of other molecular determinants of the MTBC*,* such as in RskA [17] and PhoR [18], and during the emergence of antibiotic resistant mutants [19, 20]. These point mutations have also helped establish the evolutionary relationships between several lineages and were even suggested to be useful as broad phylogenetic markers [15, 21–23].

Compared to the study of gene flow, where several bioinformatics tools exist, studying the impact of small modifications is often perilous. This kind of investigation usually involves mapping reads or genomic sequences against a close reference and then looking at the impact on the coding sequences. Tools such as snippy (https://github.com/tseemann/snippy) and snpEff [24] (used by snippy) allow this analysis efficiently. Other tools, for example kSNP3.0 [25] and the Harvest suite [26], integrate an evolutionary approach involving a phylogenetic reconstruction. However, these tools make it difficult to quickly analyze different datasets and to infer the impact of the AA changes without advanced expertise in bioinformatics. More importantly, they are impractical to identify SNPs or single amino acid polymorphisms (SAPs) that are concomitant to a specific phenotypic change that arose within a bacterial genus. The more ancestral these subtle modifications are (such as those that arise at the birth of a specific genus), the more difficult their detection will be due to time effects.

Here, we describe a new bioinformatics tool, CAPRIB (Comparative Analyses of Proteins In Bacteria), which efficiently finds through an easy-to-use graphical interface amino-acid changes that are correlated with the emergence of a phenotype. By comparing two groups of species separated by a phenotypic switch, this program can pinpoint the evolutionary events, at the genus scale, that may have been involved in this transition. This tool, whose core is in Java, uses a relational database in SQL to store raw information, allowing users to quickly change the structure of groups and save projects. In order to help users decipher the impact of AA changes, CAPRIB integrates statistical values (Grantham’s distance [27] and exchangeability score [28]) that predict the potential impact of one amino acid change for another. It is also possible to put in relation the detected AA changes and the Conserved Domain Database (CDD) of the NCBI to have an insight into the structural involvement of the permutations.

Herein, we are specifically exemplifying the application of CAPRIB using the *Mycobacterium* genus from the *Actinobacteria* phylum. This genus is an ideal candidate to study bacterial evolution through amino-acids changes because of its genotypic and phenotypic diversity. First of all, mycobacteria species can be pathogenic, commensal or saprophytic in a variety of hosts and ecological niches [29, 30]. Also, this genus was historically divided into two phenotypically-different categories, fast growers and slow growers [31]. Slow growing species require more than 7 days before colonies become visible on solid media, while rapid growing species form colonies in less than 7 days, typically within 2–5 days [32]. This phenotype was later clearly linked with phylogeny [31]. More recently, based on genomic sequences, a third pseudo-intermediate lineage was revealed [33]. These different growth rates are intrinsically linked to the evolution of mycobacteria and are the basis of their phylogenetic classification [33]. With this example, we demonstrate that CAPRIB can detect AA changes associated with a given node of evolution linked to a potential phenotypic switch (herein growth rate). As a side application, this tool can also be used, at the genus scale, to detect lineages markers (as done with DNA SNPs at the species scale) or to shed light on homoplastic characters by exploring alternative phylogenetic topologies.

## Implementation

### Development of CAPRIB

CAPRIB is developed in Perl, SQL and Java. Perl is used to filter the BLAST reports, but also to communicate with the CDD-NCBI and combine this result with the candidate protein files, ultimately generating the report in TSV format. SQL is used to build, manage and access the database. Finally, the core of CAPRIB and its GUI are in Java, mainly using the javax.swing and java.awt graphical libraries. It is designed to work on macOS, GNU/Linux and windows on a standard computer.

CAPRIB uses the results of TBLASTN (which can be generated directly with CAPRIB) in order to determine the similarity links (identical, similar or different) between the amino acids for the homologous proteins of the given dataset. Of note, we used TBLASTN (as compared to BLASTP) to avoid differences due to disparities in annotation/prediction of protein sequences from the dataset. The AA comparison information from the TBLASTN is used to generate an SQL database (see example below). Subsequently, this database can be queried to perform different operations (Table 1). The CAPRIB software and its documentation are freely available at https://github.com/BactSymEvol/Caprib.

**Table 1:** Operations available in CAPRIB

Operation	Definition	Set^**a**^
I vs D	Identical amino acids in group A that changed to a different amino acid in group B	I_A_ ∩ D_B_
IS vs D	Identical or similar amino acids in group A that changed to a different amino acid in group B	(I_A_ ∪ S_A_) ∩ D_B_
I vs S	Identical amino acids in group A that changed to a similar amino acid in group B	I_A_ ∩ S_B_
GapIn	Gaps conserved in group A	N_A_ ∩ (S_B_ ∪ D_A_)
GapOut	Gaps conserved in group B	(I_A_ ∪ S_A_∪ D_A_) ∩ O_B_
Stop codon	Amino acids in group A replaced by a stop codon in group B	(I_A_ ∪ S_A_∪ D_A_) ∩ P_B_

^a^sets of amino acid positions that are identical (I), similar (S), different (D), starting position of a gap in the reference organism (N), starting position of a gap in the compared organism (O) and the position of a stop codon (P)

### Phylogeny of mycobacteria

A dataset of species of the *Mycobacterium* bacterial genus has been assembled to optimize the quality of genomes and to be representative of species diversity (Supplementary File 1). A phylogenetic tree was created using a bioinformatics protocol published elsewhere [34]. Briefly, the 56 genome sequences (including the outgroup) were annotated using Prokka version 1.13.7 [35]. Homologous links between the translated coding sequences were found using the combination of the two algorithms COG [36] and OMCL [37] through GET_HOMOLOGUES version 20190411 [13]. The 958 gene sequences (excluding paralogs) corresponding to the softcore (sequences present in more than 95% of the genomes) were aligned using mafft version 7.407 [38]. The resulting alignments were filtered using BMGE version 1.12 [39] and concatenated in a partitioned supermatrix using AMAS [40]. The evaluation of the best-fit model of each partition and the maximum-likelihood phylogeny was done using IQ-TREE version 1.6.11 [41, 42]. The robustness of the tree was assessed by performing 10,000 ultrafast bootstraps [43]. A phylogenetic tree was also made using the nucleotide sequences of the same 958 genes and using the same protocol, with the difference that the sequences were codon aligned using TranslatorX version 1.1 [44].

### Analysis with CAPRIB

Three databases were constructed: one using *M. tuberculosis* H37Rv (slow growing) as the reference, one with *M. gilvum* Spyr1 (fast growing), and one with *M. abscessus* ATCC 19977 (basal fast growing). The comparisons were generated using TBLASTN version 2.9.0+ [45]. The same 56 genomes utilized for the presented phylogeny were also used but *M. leprea* and *M. lepraemurium* were excluded since they have a specific well-described evolution by reductive genomics and gene decay with respectively 41% [46] and 30% pseudogenes [47]. The identity threshold for considering two sequences as homologues was determined by calculating the 10th percentile median for all BLAST results for a given reference. Although analyses could be done without any threshold, we recommend this empirical threshold to minimize the likelihood of false-positive assignments due to low-level similarity as determined previously [8]. This permitted determining a minimum of 40% of protein similarity using *M. tuberculosis* H37Rv (i.e. we extracted AA changes only for proteins harboring > 40% similarity score with all species in the dataset), 35% for *M. gilvum* Spyr1, and 33% for *M. abscessus* ATCC 19977 as references. The functional categories for some proteins of interest were determined using the eggNOG 5.0.0 database [48]. For comparative analysis between fast-growing and slow-growing mycobacteria, only the species after node 2 in Fig. 2 were considered for the slow category (excluding *M. terrae* clade species because of their intermediate phenotype) and all species before node 1 in Fig. 2 (excluding outgroup species) were grouped in the fast category. Most of the AA changes have been verified using standard alignments.

## Results

### Description of CAPRIB main functions

**Fig. 1:**
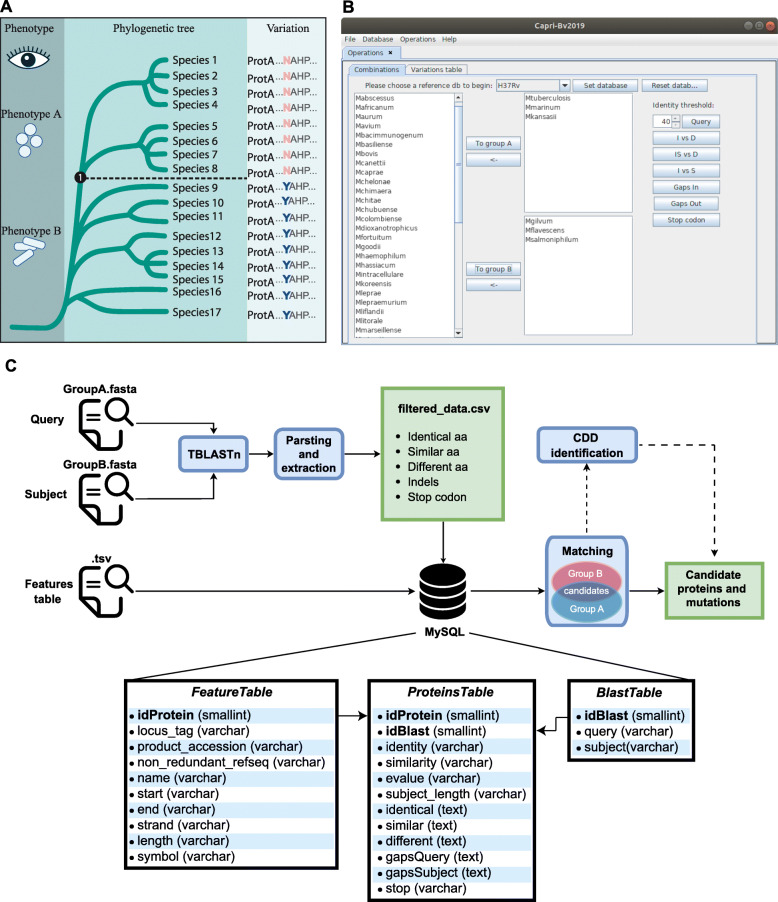
**a** Schematization of the evolutionary approach used by CAPRIB. **b** CAPRIB main interface showing the possibility of defining groups and changing parameters. **c** Diagram of CAPRIB workflow and SQL database structure

In order to investigate the amino acid changes that may have been involved in the evolution of a group of organisms and in the emergence of a new phenotype (see Fig. 1a), we have created CAPRIB, a tool with an easy-to-use graphical interface (Fig. 1b). To make CAPRIB accessible to a maximum number of users, it only requires some dependencies that are often already installed on biologists’ computers (Java, MySQL, PERL and BLAST+). It has also been designed to work on most operating systems (Windows, macOS and GNU/Linux). The architecture of CAPRIB, relying on relational databases (Fig. 1c), allows users to create projects and to change parameters easily for a given database, without having to redo the BLAST searches. A summary of the results can be visualized directly through CAPRIB while complete detailed results are available in a CSV file, compatible with spreadsheet tools. To guide users through all of the AA changes found by CAPRIB, the latter integrates an interface allowing to check if the permutations are in conserved domains according to the NCBI CDD database. In addition, scores (Grantham’s distance [27] and exchangeability score [28]) are associated with each of the AA changes which help assessing the potential impact of the permutations on the structure of the proteins.

#### The SQL database

The user is performing TBLASTN (with BLAST+ using the Java interface) of protein sequences from reference (herein *M. tuberculosis* H37Rv a slow-growing species, *M. gilvum* Spyr1 a rapid-growing bacteria, and *M. abscessus* ATCC 19977 a basal fast-growing species) against genomic sequences of other species from the studied genus (herein 51 mycobacteria species and the three outgroup bacteria). Subsequently, the user is using the results of these comparisons to feed the database. The database is structured as shown in Fig. 1c with a table of general features from the reference organism (such as the name of proteins, locus tags, accession numbers), a second table with the blast information (query and subject names) and a third table that comprises the information extracted from the BLAST file. For this latter, Perl is used to parse and extract results from the BLAST report. The originality of this tool is that it uses the information contained in the BLAST results and classifies each compared AA as identical, similar, or different in regard to the reference in a third SQL table (see Fig. 1c). CAPRIB also stores information of gaps (insertion of AAs or deletion of AAs) and AAs that changed to stop codon. This unique property allows to store information of comparisons of all proteins from the reference against all genomic sequences using minimal computational requirement and thus to use standard computers.

#### Comparisons

The user can query the database according to different evolutionary strategies (Table 1). The first, I vs. D, identifies positions with identical AAs in group A, but different in group B. The main objective of this strategy is to find positions with high conservation pressure in group A, and with a relaxed pressure in group B. The 2nd strategy, IS vs. D, is less stringent than the first one and allows including positions with similar AAs in group A. The 3rd strategy, I vs. S, makes it possible to identify the slightly subtler differences of the protein structure since it indicates the positions with identical AAs in group A and similar to group B. The 4th and 5th strategies allow finding the gaps preserved in group A (GapIn) or B (GapOut), respectively. Finally, the sixth strategy (Stop codon) makes it possible to identify stop codons present exclusively in group A, and thus to infer the potential truncations of proteins.

#### Tools to infer the severity of AA changes

Once the user has performed comparisons, some tools are integrated to facilitate the classification of AA changes in function of their predicted effect. Two scores that are predictors of the effect of the substitution of one AA to another are implemented in CAPRIB. The first one is the Grantham distance [27] that takes into account three parameters of AAs, such as composition, polarity, and molecular volume, permitting to compare the propensity of AAs to permute based on their biochemical properties. This table of one-by-one AA comparison correlates with protein residue substitution frequencies [27]. The second score is based on a study from Yampolsy et al. (2005) that has experimentally measured the Experimental Exchangeability (EX) of each AA by another one by replacing around 10,000 AAs in 12 proteins [28] which also generated a table of scores for AAs exchangeability. Of note, in this case the score is inversely proportional to the ease of exchangeability. They have also measured the overall scores of specific AA exchangeability (Exchangeability as a source: EXsrc or as destination: EXdest). In CAPRIB, these scores are indicated in the results file next to the AA change (ex: 75 = A/H:86:301:EXsrc = 312:EXdest = 290, herein the AA number 75, which is an alanine in group A, is replaced by a histidine in this query species from group B. This change has a Grantham score of 86 and an Ex score of 301. The EXsrc value of the A and the EXdest of the H are also indicated).

We have also implemented an option that links the result file with CDD (Conserved Domain Database) of NCBI [49] as described in the help of CDD (https://www.ncbi.nlm.nih.gov/Structure/cdd/cdd_help.shtml#BatchRPSBWebAPI_GETorPOST). We have used the class CddNcbi which starts the script ccd.pl, collects results and fuses them with the list of AA changes to produce a TSV file that can be directly opened in CAPRIB or online on the CDD website. With this option, the users can verify if permutated AAs are located at specific positions of the proteins, such as conserved domains.

### Validation and comparison of CAPRIB with a gold standard method

**Fig. 2:**
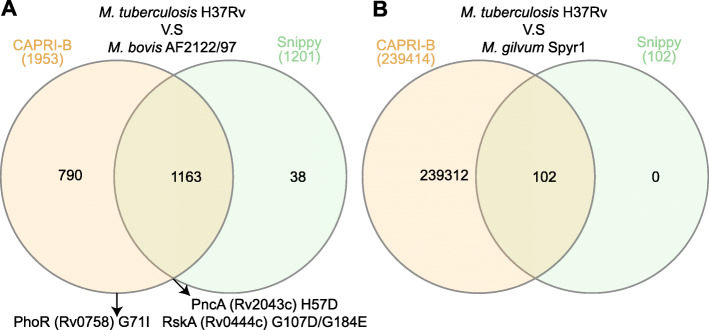
Venn diagrams showing the number of AA changes found between **a** *M. tuberculosis* H37Rv and *M. bovis* AF2122/97 and **b** *M. tuberculosis* H37Rv and *M. gilvum* Spyr1 by CAPRIB and Snippy

The accuracy of CAPRIB has been validated by comparing it to the Snippy tool (https://github.com/tseemann/snippy), which automates a gold-standard bioinformatics procedure commonly used to compare bacterial genomes. Since Snippy does not natively allow group comparisons, we first compared two organisms using *M. tuberculosis* H37Rv against *M. bovis* AF2122/97 (Supplementary File 2). The differences between the two members of the *Mycobacterium tuberculosis* complex (MTBC) are also well described [50]. As illustrated in Fig. 2a, the majority of AA permutations were found by both CAPRIB and Snippy, which was expected since the two species are very close at the genetic level [51]. However, several AA changes were found only by CAPRIB, while few were only found by Snippy. Mutations with a functional impact, well known in the literature and verified experimentally, such as PhoR (Rv0758) = G71I [18], PncA (Rv2043c) = H57D [52], or two mutations in RskA [17] were sought to validate CAPRIB. All of these mutations were correctly identified by CAPRIB, while Snippy misses the PhoR AA change.

A thorough investigation by sequence alignments revealed that several mutations only found by CAPRIB are in degenerated regions of proteins. Snippy generates fake sequencing reads of 250 bp and then maps them on the reference genome sequence with BWA mem, calls the mutations with freebayes and determines the effect of the mutations with snpEff. Although this procedure is commonly used, it requires closely related bacterial strains since it makes a comparison between short nucleotide sequences and therefore does not allow optimal alignment between derived sequences. CAPRIB, compared to Snippy and other methods, uses the TBLASTN tool, which allows comparisons between protein sequences and generates a longer alignment, and is therefore more precise in derived regions. To further illustrate this phenomenon, we compared *M. tuberculosis* H37Rv against *M. gilvum* Spyr1, i.e. two distant species (Fig. 2b and Supplementary File 3). Very few mutations were found with Snippy compared to CAPRIB and, more importantly, all the mutations found by Snippy were also found by CAPRIB. To our knowledge, no other tool is able to perform AA comparisons and extract substituted AA between two groups. Consequently, CAPRIB is a functional, unique and powerful tool allowing to study AA changes between groups of evolutionary distant bacteria, like those composing a bacterial genus.

### Use of CAPRIB to monitor the phylogenetic landscape and assess putative abnormality

**Fig. 3:**
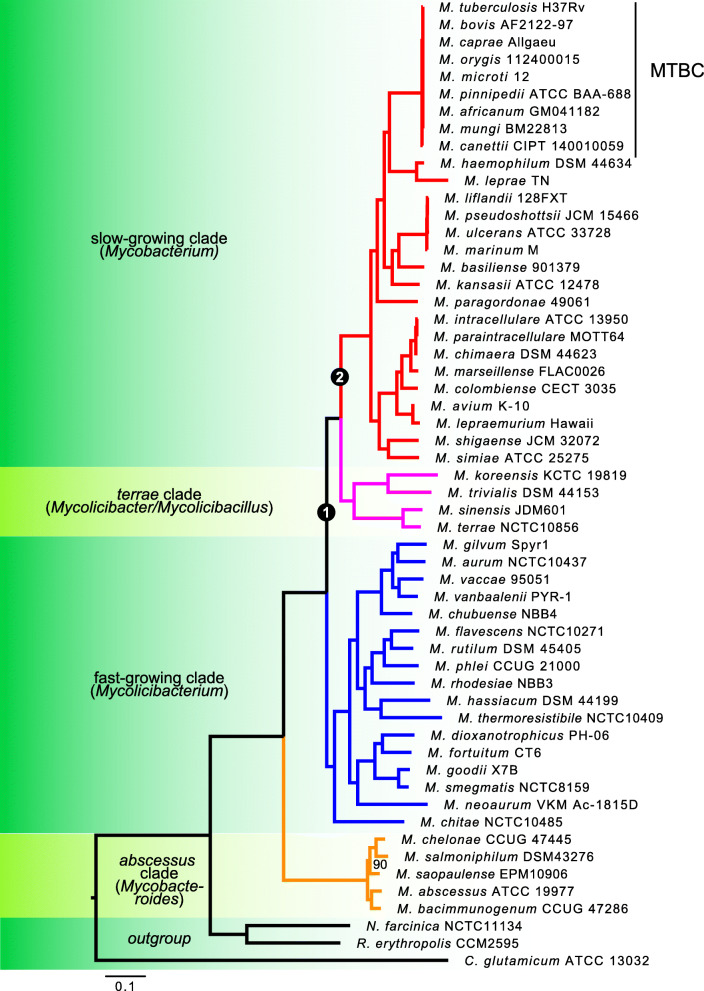
A phylogenetic tree of the *Mycobacterium* genus based on softcore sequences, as described in the implementation section. Clades containing the majority of slow, fast and intermediate growth species are represented in red, blue and pink, respectively. The *abscessus*-clade is represented in orange. The different organisms were also classified according to a study that separated the genus *Mycobacterium* into five genera [54]. The different nodes specifically investigated by this study are indicated on the tree. The set of bootstraps values are at 100, with the exception of some nodes where the values are indicated

Mycobacteria were historically separated into two large phylogenetic groups that were correlated with their growth rate [31]: the slow-growing and fast-growing lineages. Recently, a third group (*M. terrae* complex) has been revealed to be an intermediate (in terms of phylogeny but also in terms of growth phenotype) between the slow and fast-growing mycobacteria [33, 53]. The fact that mycobacteria can be separated phylogenetically based on a phenotype (growth rate) makes it a model of choice to be investigated with CAPRIB. Three databases were constructed using 53 genomes of mycobacteria with genomes of good quality (Supplementary File 1) and *M. tuberculosis* H37Rv (slow grower), *M. gilvum* Spyr1 (fast grower), and *M. abscessus* ATCC 19977 (basal fast grower) as references. The Fig. 3 presents the phylogenetic relations of species composing this dataset, which is in accordance with previously published phylogenies [33, 53]. A second phylogeny was made from nucleotide sequences as indicated in the implementation section. Both phylogenies are highly identical, with the exception of a node inside the MTBC (data not shown).

Defining groups is crucial in order to achieve optimal results with CAPRIB. Of note, gold standard tools to infer phylogenetic reconstruction by maximum-likelihood, such as IQ-TREE [42] and RAxML [55, 56] could eventually bias grouping as they force strict bifurcating trees and do not allow polytomy. The fact that *M. terrae* clade species are considered as intermediates between slow and fast growers can cause a problem in the definition of groups and thus in the different AA changes found to explain the phenotype (i.e., growth difference). The molecular phylogeny carried out for the present study reveals the grouping of this clade among the slow growers, a result also found by several other studies [33, 54, 57]. However, the position of this clade has already been shown to be unstable by a seven genes multilocus-based phylogenetic study [58].

**Fig. 4:**
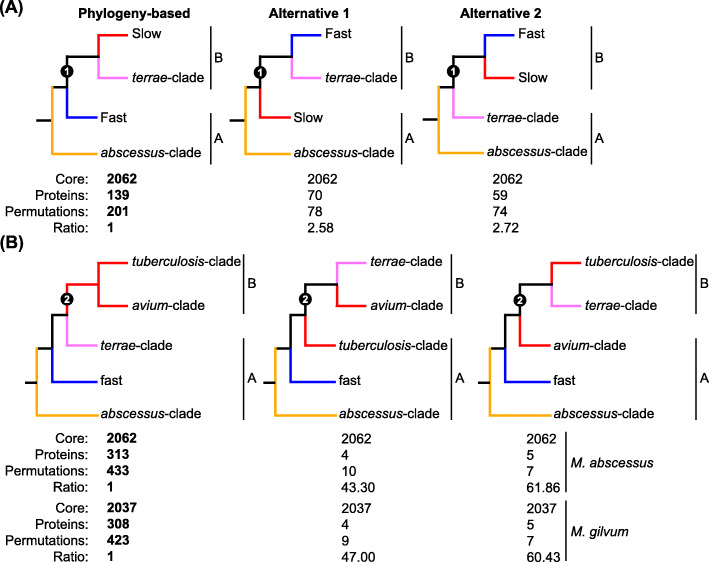
Analyzes of different phylogenetic topologies of mycobacteria using CAPRIB to validate **a** the position of *M. terrae* clade in relation to the slow and fast growing mycobacteria and **b** the position of *M. terrae* clade among the slow growing mycobacteria. The column “Phylogeny-based” is established via Fig. 3, while the other two columns represent alternative topologies. For each of the topologies, the number of core proteins, the number of proteins with AA changes, the number of AA changes, and finally the AA changes ratio of the phylogeny-based topology to that analyzed are given. Results in bold indicate the topology with the most markers for each group of trees

Since CAPRIB can find markers specific to different groups, we challenged the phylogenetic position for this clade as shown in Fig. 4. In this context, it is usually expected that the number of homoplastic sites (AAs that are shared by a set of species, not present in their common ancestor and that originated independently as a result of convergent evolution) will be inferior as compared with vertically derived sites from the last common ancestor. Using *M. abscessus* ATCC 19977 as a reference, the number of AA changes was determined using CAPRIB for the topology derived from the phylogeny obtained in Fig. 3 but also for the two other alternative topologies that also correspond, if the phylogeny is correct, to homoplastic sites (Fig. 4). The topology of node 1 obtained by molecular phylogeny in Fig. 3, namely that the fast-clade is basal to the *M. terrae*-clade cluster with the slow growers, is the one with the most markers (201 conserved permutations) (Fig. 4a). On the contrary, the alternative topologies have fewer conserved permutations (78 and 74).

In a second step, since it was possible to confirm that the terrae-clade shares a common ancestor with slow growers, we further investigated the relationships between these two groups (node 2 in Fig. 3) (Fig. 4b). Since the fast growing phenotype is paraphyletic, two databases were used, one with *M. abscessus* ATCC 19977 as the reference and the other with *M. gilvum* Spyr1, allowing to assess the topologies using references from both fast groups. This also permitted to exclude any effect due to the choice of reference. Again and as expected, the topology with the most markers is the one that corroborates the molecular phylogeny, namely that the terrae-clade is basal to the slow-growing-species group, composed of the *tuberculosis* and *avium* clades, which share a common ancestor. Results obtained with the two databases are also very similar.

### Specific example showing the macroevolution from fast to slow-growing mycobacteria

In the context of the stepwise evolution of the slow-growing mycobacteria with *M. terrae*’s clade presenting an intermediate growth phenotype, we performed the analysis by ignoring the latter group. In this case, AA changes found by excluding this clade therefore represent the changes that happened before the divergence of the slow+*M. terrae* clade from fast-growing mycobacteria (node 1) in addition to the changes that happened after the divergence of the slow from the *M. terrae* clade (node 2). Also *M. leprea* and *M. lepraemurium* were excluded since their genomes harbor a high number of pseudogenes [46, 47]. Using a cutoff of 40% identity, 1773 proteins common to all genomes were found using *M. tuberculosis* H37Rv as a reference (Supplementary File 4) and 2122 using *M. gilvum* Spyr1 with a cutoff of 35% identity (Supplementary File 5).

Using the slow-growing species *M. tuberculosis* H37Rv, CAPRIB has allowed us to identify 1462 conserved amino acids that are different in fast-growing species. These AA changes are distributed among 709 of the 1773 genes found in *M. tuberculosis* H37Rv (Supplementary File 4). Considering AA changes with a Grantham’s distance greater than 100 and an exchangeability score of less than 250, only 185 (~ 12.6%) have a high chance of altering protein function, the rest being more conservative changes (such as A/G, G/A, V/A, A/V). Similarly, using the fast-growing species *M. gilvum* Spyr1, 1092 AA changes were found to be distributed among 567 proteins. Of these changes, 129 (~ 11.8%) have a Grantham’s distance greater than 100 and an exchangeability score of less than 250.

**Fig. 5:**
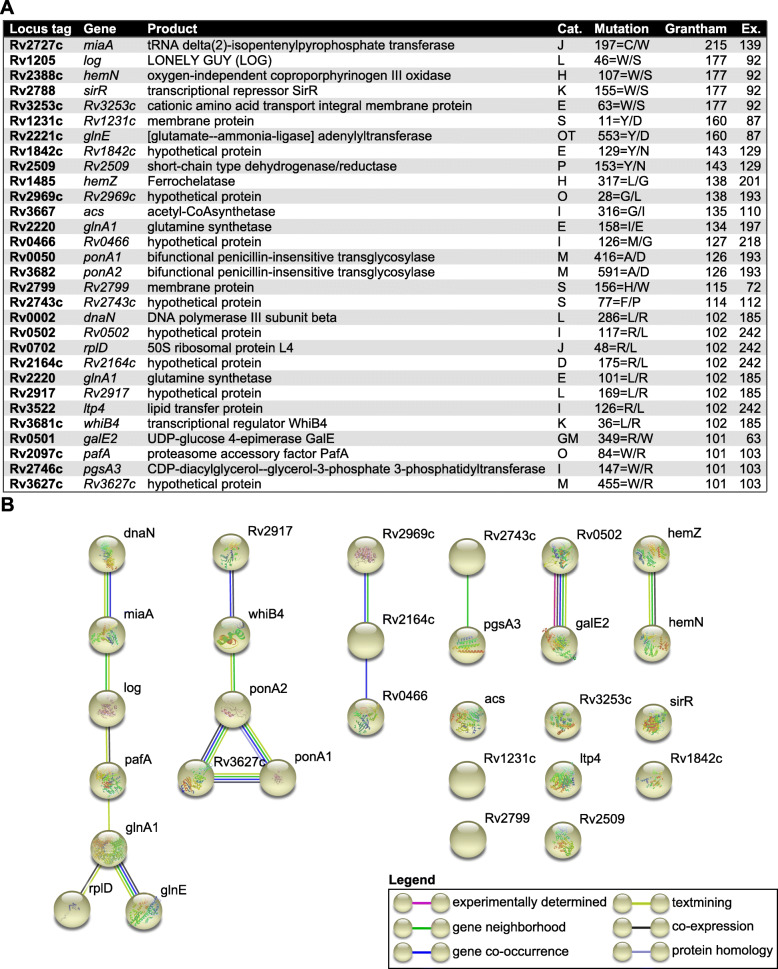
**a** AA changes between the slow and fast-growing mycobacteria, but conserved in each group. **a** STRING analysis showing putative relationships between proteins from the “**a**” panel

The lists of mutations found using *M. tuberculosis* H37Rv and *M. gilvum* Spyr1 with a Grantham’s distance greater than 100 and an exchangeability score of less than 250, were crossed to find the different permutations between slow and fast growers, but that are identical within the groups, such as schematized in Fig. 1. This highly stringent analysis permitted to find 30 mutations having a high conservative pressure in both groups but that are drastically different between groups (Fig. 5a). We later investigated the biological functions of the proteins containing the mutations and the putative pathways that could link them through a STRING analysis (Fig. 5b). It was interesting to note that this analysis can highlight some hotspots or pathways that could have evolved in the ancestor of slow growing mycobacteria after its divergence with rapid growing mycobacteria, such as the two proteins GlnE and GlnA1 encoded by neighbor genes, or the PonA1, PonA2 and WhiB4 proteins (with PonA2 and WhiB4 encoded by neighbor genes). In addition to these, it was possible to shed light on three proteins (Rv2727c:MiaA; Rv1205:LOG; Rv2097c:PafA) involved in cytokinin production [59], a molecule related to a phytohormone influencing plant growth and development [60]. Again, these three genes are often found in the same locus [61]. Interestingly, using CDD link within CAPRIB, we realized that a recent study solved the structure of the LOG protein from *Corynebacterium glutamicum* and found that the equivalent residue is involved in AMP binding [62]. The fact that some hotspots of evolution could be revealed emphasizes the strategic fit of the methodology developed and that it could be applied in multiple evolutionary contexts.

## Discussion

The stepwise adaptation of bacteria occurs through genetic alterations, in which only permissive changes are selected at each of these steps. By deciphering the adaptive mechanisms or pathogenesis emergence of these bacteria, we will not only be able to find crucial information on the bacterial physiology, but also approaches for the treatment and diagnosis of infections. The high availability of sequencing technologies now allows genomes to be investigated on an unprecedented scale. Numerous tools focus on the detection of genetic events just before the speciation of a pathogen, leaving behind step-wise ancestral events at different nodes of evolution (including the ones not directly linked to pathogens’ speciation) that have drastic consequences on the pathogens as we know them today (what could be called the “butterfly effect”). To illustrate this concept, we could cite the mycomembrane, which has evolved long before the host-adaptation of mycobacteria and is now playing a key role in *M. tuberculosis* pathogenesis [63]. The CAPRIB tool described in this study allows users to extract, at the genus scale, drastic AA changes that are concomitant to a phenotypic switch between two bacterial groups. Rich from this knowledge, biologists could now focus their attention on these key residues and on the impact of these changes using molecular microbiology approaches. One of CAPRIB’s goals is to make data analysis simple, so it integrates a user-friendly graphical interface and relies on an SQL relational database, allowing comparisons between genomes to be saved. In addition, to maximize accessibility to CAPRIB, it has been designed to work on the majority of operating systems (Windows and UNIX-like).

During our software optimization, we realized that several AA changes in many proteins were detected but the majority of them were conservative. This is why CAPRIB integrates two scores (Grantham’s distance and exchangeability score) for each amino acid change, allowing the user to infer the potential impact of the detected changes. In addition, CAPRIB allows to connect the list of AA changes with the NCBI CDD database, making it possible to check whether the permutations are in conserved domains. However, no absolute rule can infer with certainty the impact that a mutation may have on the functionality of a protein and on the network of interaction with other biological molecules. Comprehensive bioinformatics analyses, such as protein modeling and molecular dynamics, can help verifying a mutation’s impact with greater certainty. Also, other analyzes complementary to those proposed by CAPRIB could also be carried out by users. For example, CAPRIB often detects several AA changes in the same protein, in which case the user could investigate whether these sites co-evolved using SpiderMonkey, a tool using Bayesian graphical models [64]. Finally, some users might be interested in differentiating the AA changes found by CAPRIB that arose from neutral selection from those under positive selection to better understand the evolutionary forces that have motivated the evolution of proteins. Several tools to perform this type of analysis, including co-evolution of sites, are available in the Datamonkey web server [65].

The study of AA polymorphism profiles with CAPRIB requires some essential prerequisites in order to obtain optimal results. Among these, there is, of course, the quality of the dataset. The more complete a dataset is, which should be representative of the diversity of the bacterial groups studied, the more powerful the results will be. This obviously includes having a robust molecular phylogeny to properly assign the species studied to different groups. This is why a core-genome approach, with several phylogenetic markers, has been favored in this study compared to a 16S phylogeny, that is faster to perform, but often less accurate [66]. Moreover, since CAPRIB makes it possible to analyze the AA changes finely, it goes without saying that it is essential to have sequences of good quality. Fortunately, sequencing errors are often random and the allocation of bases is getting better. Also, it is ideal to compare groups with similar patristic distances (same length of branches in the phylogeny). Although not essential, this makes it possible to minimize the number of false positives.

In the course of our study, we also realized that CAPRIB could be used to question phylogeny reconstructions similarly than what has been done with DNA SNPs to resolve species phylogeny. Our results concerning AA changes clarified the divergence of the slow, rapid and intermediate mycobacterial lineages, an issue still debated in the literature [33, 53]. Although the results obtained with CAPRIB corroborate the topology from molecular phylogeny, several homoplastic sites support alternative topologies. These homoplastic sites may have blurred true evolutionary signals when lower resolution approaches were used in the past [58]. The detection of lineage-specific AA changes, at the genus scale, can therefore be of great use for determining markers in the context of detection and identification strategies.

On the other side, we used CAPRIB to shed light on some of the genetic events potentially involved in the emergence of the slow-growing lineage that comprises the majority of pathogenic *Mycobacterium*. Of note, other events such as gene insertions or deletions may also have influenced this process, but this was not the scope of CAPRIB as this has already been investigated [8, 67, 68]. It is interesting to note that several AA changes in proteins involved in the bacterial membrane have been found. This is consistent with the fact that this organelle is at the boundary with the environment/host and is therefore an evolutionary hotspot. This is especially true in mycobacteria that have a unique cell envelope among bacteria, the genesis of which is still debated [11]. AA permutations in regulators have also been observed. These proteins have the ability to change the level of expression of several genes and thus have the potential to greatly impact global protein networks, resulting in important phenotypic differences. Finally, we shed light on the evolution of numerous hotspots such as the mycobacterial cytokinin pathway, concomitant to the emergence of slow-growing mycobacteria. There are only a few studies on the role of this family of molecules that is similar to a phytohormone influencing plant growth and development [60]. Some have shown that it can influence signaling in *M. tuberculosis* [69] whereas others have discovered that cytokinin accumulation is conditionally deleterious as it can lead to an aldehyde breakdown product that kills mycobacteria in the presence of nitric oxide produced by macrophages [59]. It remains to be tested if evolution has sacrificed, in a biological tradeoff, the growth speed of the slow-growing mycobacteria by altering, for example, the cytokinin pathway, in order to allow a better survival in macrophages or other types of macrophage-like cells such as amebae.

## Conclusions

We created a new bioinformatics tool, CAPRIB, which can identify key amino acid changes that are concomitant with a phenotypic divergence at the genus scale. This can be applied for numerous studies driven by an evolutionary approach such as antibiotic resistance acquisition, cell-shape change or pathogenesis emergence among others. To highlight the usefulness of this software, we performed a stringent analysis (non-exhaustive) to pinpoint some of the AA changes that are concomitant with the slow-growing lineage birth inside the *Mycobacterium* genus. This tool could also potentially identify certain key proteins in different biological processes whose functions could then be validated experimentally. As previously done with *M. tuberculosis*, the current approach to assign biological functions of proteins usually involves the generation of a transposon random insertion mutant library, followed by screening to identify genes, phenotypes and essentiality [70–73]. Although this approach allows to determine the functions of proteins, it remains very laborious. As shown by the present study, CAPRIB provides a more targeted approach to identify certain candidate proteins or pathways that have been intensively reworked during evolution at the genus scale, and that would justify efforts for experimental studies.

## Availability and requirements

**Project name:** CAPRIB

**Project home page:** https://github.com/BactSymEvol/Caprib

**Operating systems:** Windows, MacOS and UNIX-like operating systems

**Programming language:** Java, Perl

**Other requirements:** Java, MySQL, PERL, BLAST+ and XAMP (Windows and Linux: 7.3.0–0; MacOS: 5.6.39). All of the dependencies are provided with the software in a ready-to-use virtual machine, available in the GitHub.

**License:** GPLv3

**Any restrictions to use by non-academics:** No

## Supplementary Information

**Additional file 1.** Genome sequences used for the molecular phylogeny.

**Additional file 2 **Comparison between *M. tuberculosis* H37Rv and *M. bovis* AF2122/97 using CAPRIB and Snippy.

**Additional file 3 **Comparison between *M. tuberculosis* H37Rv and *M. gilvum* Spyr1 using CAPRIB and Snippy.

**Additional file 4 **Results of CAPRIB using *M. tuberculosis* H37Rv as reference.

**Additional file 5 **Results of CAPRIB using *M. gilvum* Spyr1 as reference.

## Data Availability

The CAPRIB software and its documentation are freely available for Windows and UNIX-like operating systems in the github repository, at https://github.com/BactSymEvol/Caprib. Sequence information used by this study is available in Supplementary File 1. The authors confirm all supporting data, code and protocols have been provided within the article or through supplementary data files.

## Abbreviations

AA: Amino Acid
CAPRIB: Comparative Analyses of Proteins In Bacteria
CDD: Conserved Domain Database
EX: Experimental Exchangeability
GUI: Graphical User Interface
HGT: Horizontal Gene Transfer
MTBC: *Mycobacterium tuberculosis* Complex
SAP: Single Amino-acid Polymorphism
SNP: Single Nucleotide Polymorphism
SQL: Standardized Query Language
